# Supplementary material for: Autoimmune Effect of Antibodies against the SARS-CoV-2 Nucleoprotein
Source: Viruses. 2022 May 25;14(6):1141. doi: 10.3390/v14061141 (PMC9228376; doi:10.3390/v14061141)
Supplement: Supplementary file 1 [file viruses-14-01141-s001.zip › Supplementary Materials.pdf]

Sample information

Demographic and clinical features distributions and bar plots by the severity of the disease course, defined according to the CT scans.

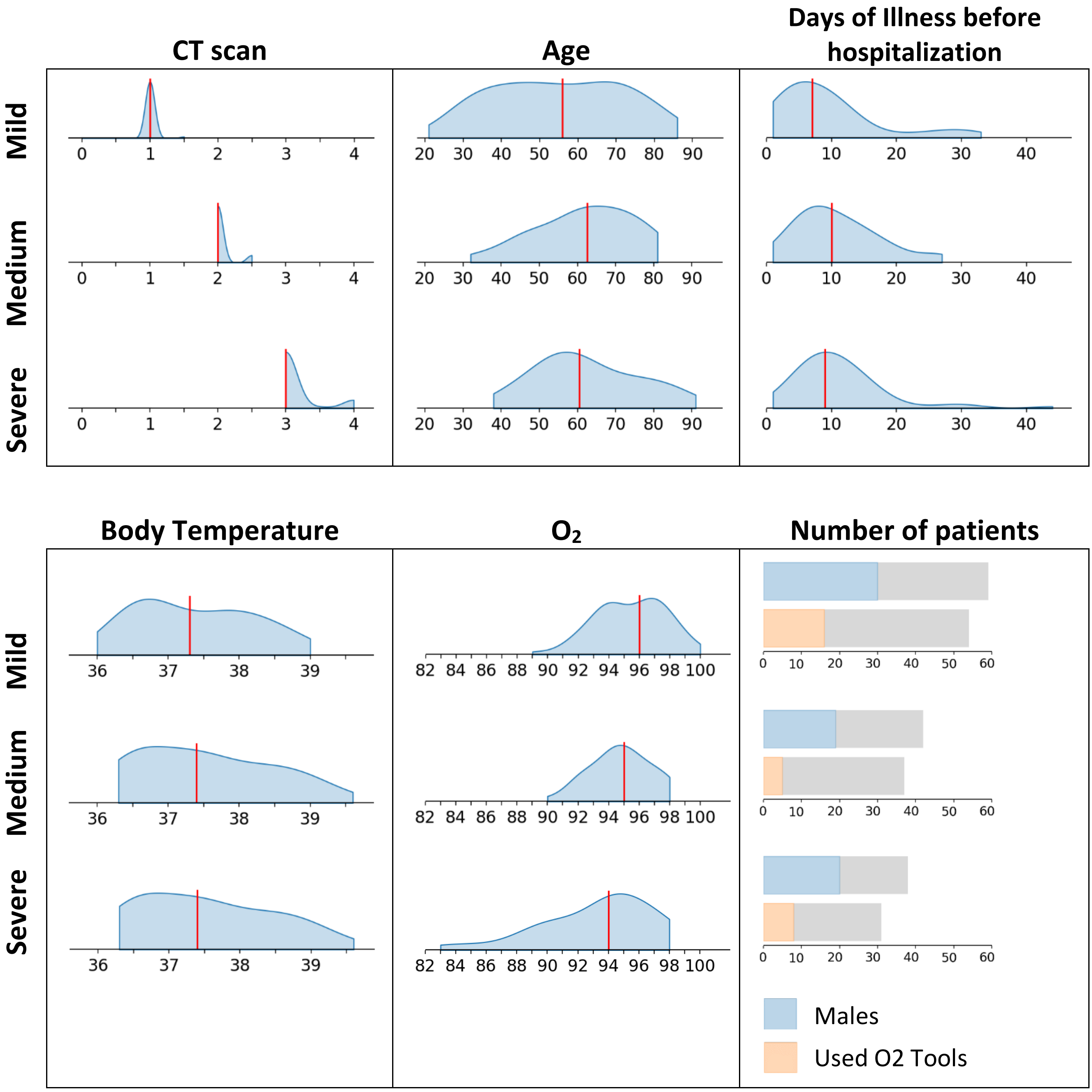

The red vertical lines represent the median values.

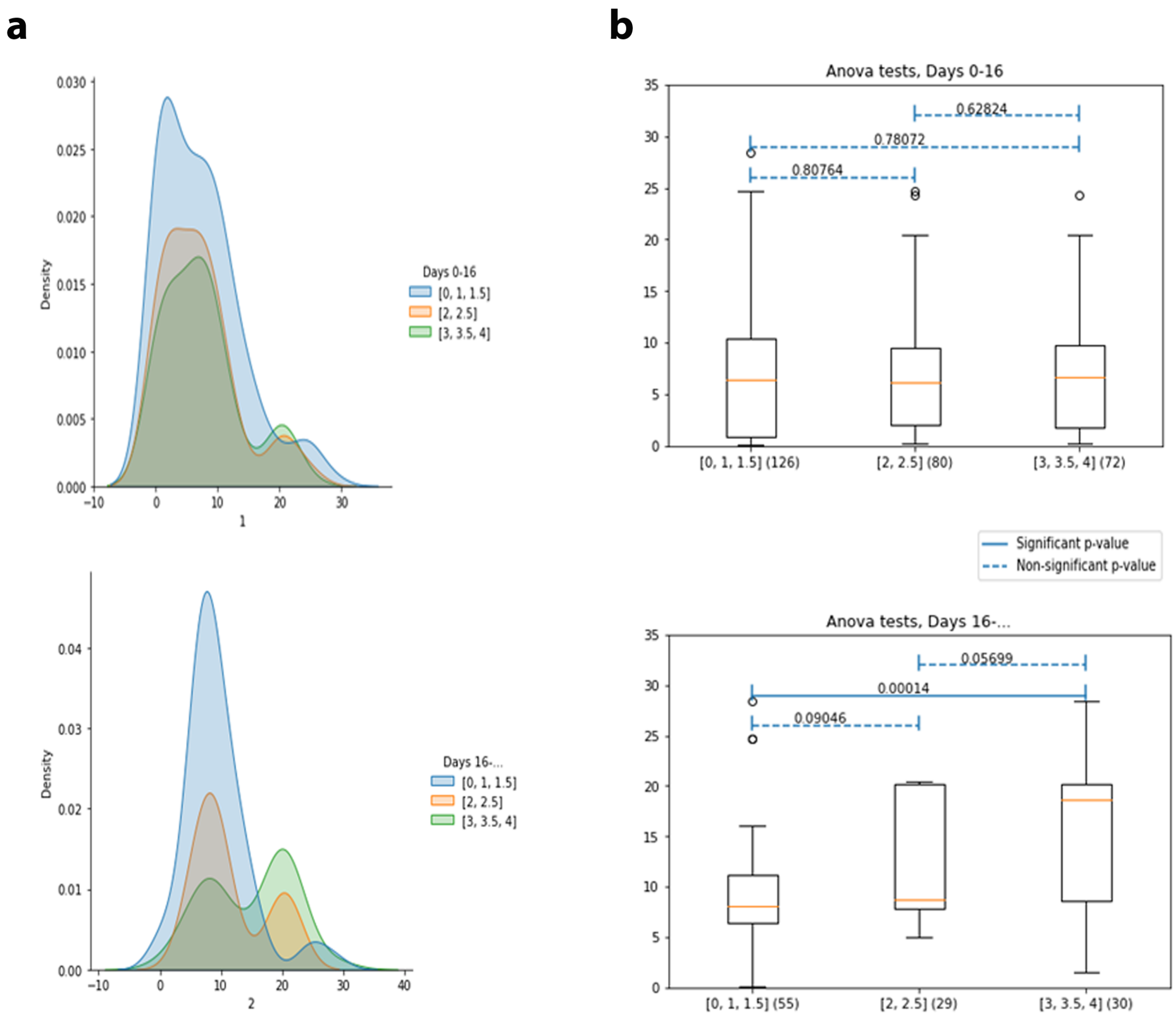

Figure S1. Main plots showing growth / peak / decay zones of antibodies to the N protein conformational epitopes according to ELISA data. (a) The plots show the distributions of antibodies levels for different severity groups of patients (0, 1, 1.5 – the mild severity; 2, 2.5 – the medium severity disease; 3, 3.5, 4 – the most severe course of disease) on 3 different disease stages: the growth period (days 0-16), the following stage of disease (days 16-…). **(b)** The boxplots demonstrate difference of antibodies levels for 3 severity groups (0, 1, 1.5 – the mild severity; 2, 2.5 – the medium severity disease; 3, 3.5, 4 – the most severe course of disease). Above the boxplots p-values of pairwise ANOVA tests are written. The segments designate for which pair the p-value is given. Dashed lines imply insignificant differences between severity groups, solid lines – significant differences

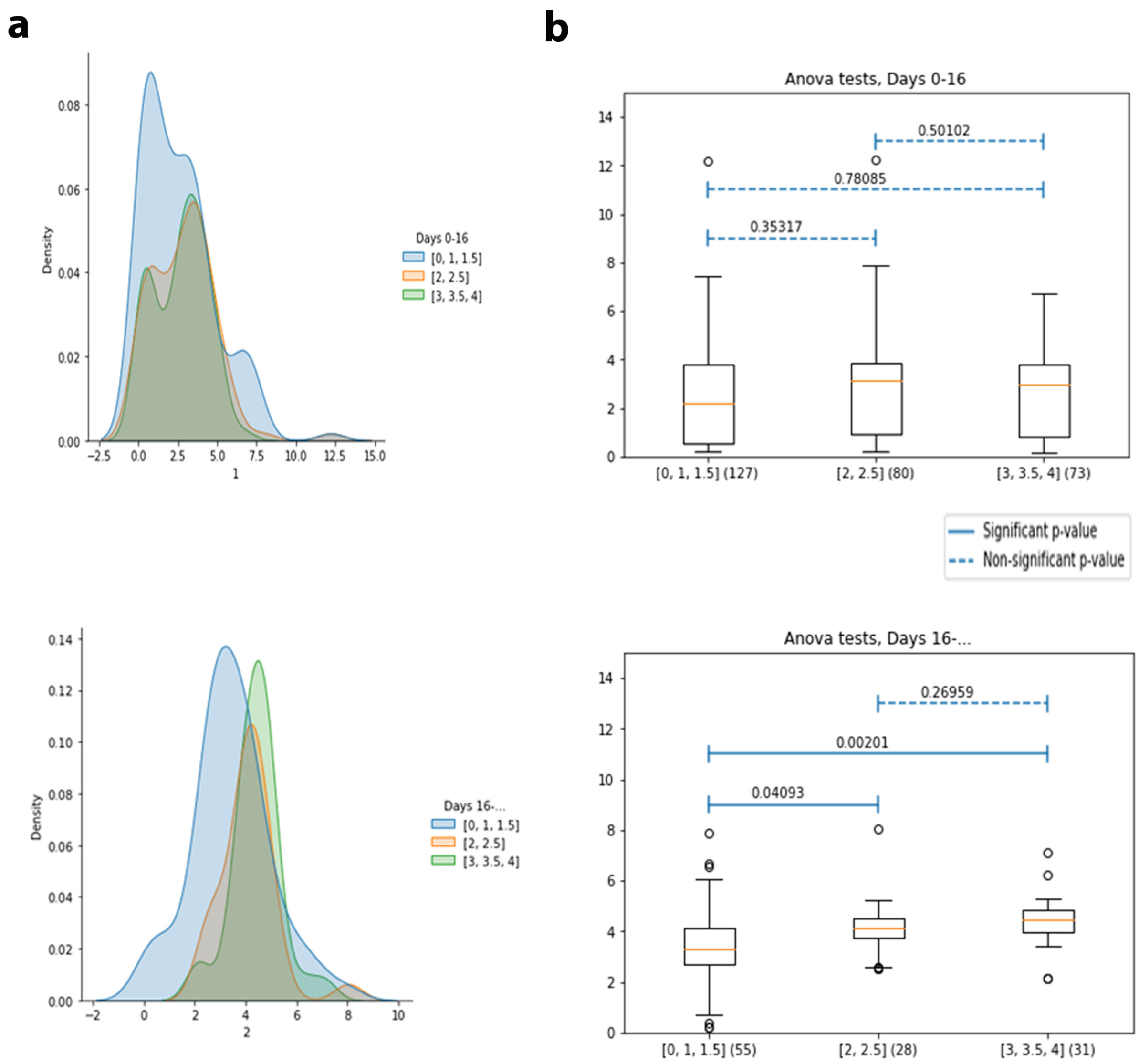

Figure S2. Main plots showing growth / peak / decay zones of antibodies to the RBD conformational epitopes according to ELISA data. (a) The plots show the distributions of antibodies levels for different severity groups of patients (0, 1, 1.5 – the mild severity; 2, 2.5 – the medium severity disease; 3, 3.5, 4 – the most severe course of disease) on 3 different disease stages: the growth period (days 0-16), the following stage of disease (days 16-…). **(b)** The boxplots demonstrate difference of antibodies levels for 3 severity groups (0, 1, 1.5 – the mild severity; 2, 2.5 – the medium severity disease; 3, 3.5, 4 – the most severe course of disease). Above the boxplots p-values of pairwise ANOVA tests are written. The segments designate for which pair the p-value is given. Dashed lines imply insignificant differences between severity groups, solid lines – significant differences

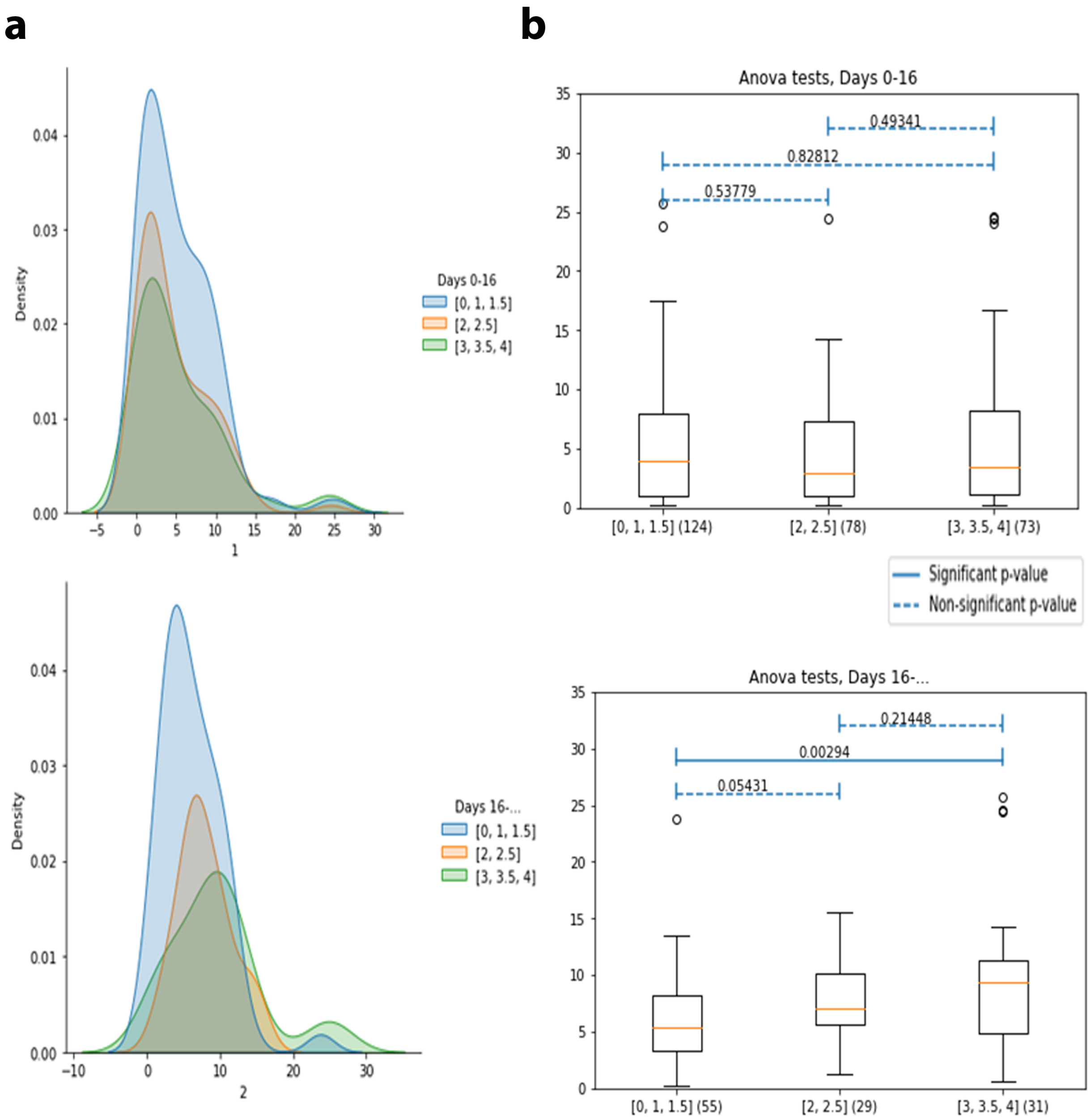

Figure S3. Main plots showing growth / peak / decay zones of antibodies to the S2 domain of S protein conformational epitopes according to ELISA data. (a) The plots show the distributions of antibodies levels for different severity groups of patients (0, 1, 1.5 – the mild severity; 2, 2.5 – the medium severity disease; 3, 3.5, 4 – the most severe course of disease) on 3 different disease stages: the growth period (days 0-16), the following stage of disease (days 16-…). **(b)** The boxplots demonstrate difference of antibodies levels for 3 severity groups (0, 1, 1.5 – the mild severity; 2, 2.5 – the medium severity disease; 3, 3.5, 4 – the most severe course of disease). Above the boxplots p-values of pairwise ANOVA tests are written. The segments designate for which pair the p-value is given. Dashed lines imply insignificant differences between severity groups, solid lines – significant differences

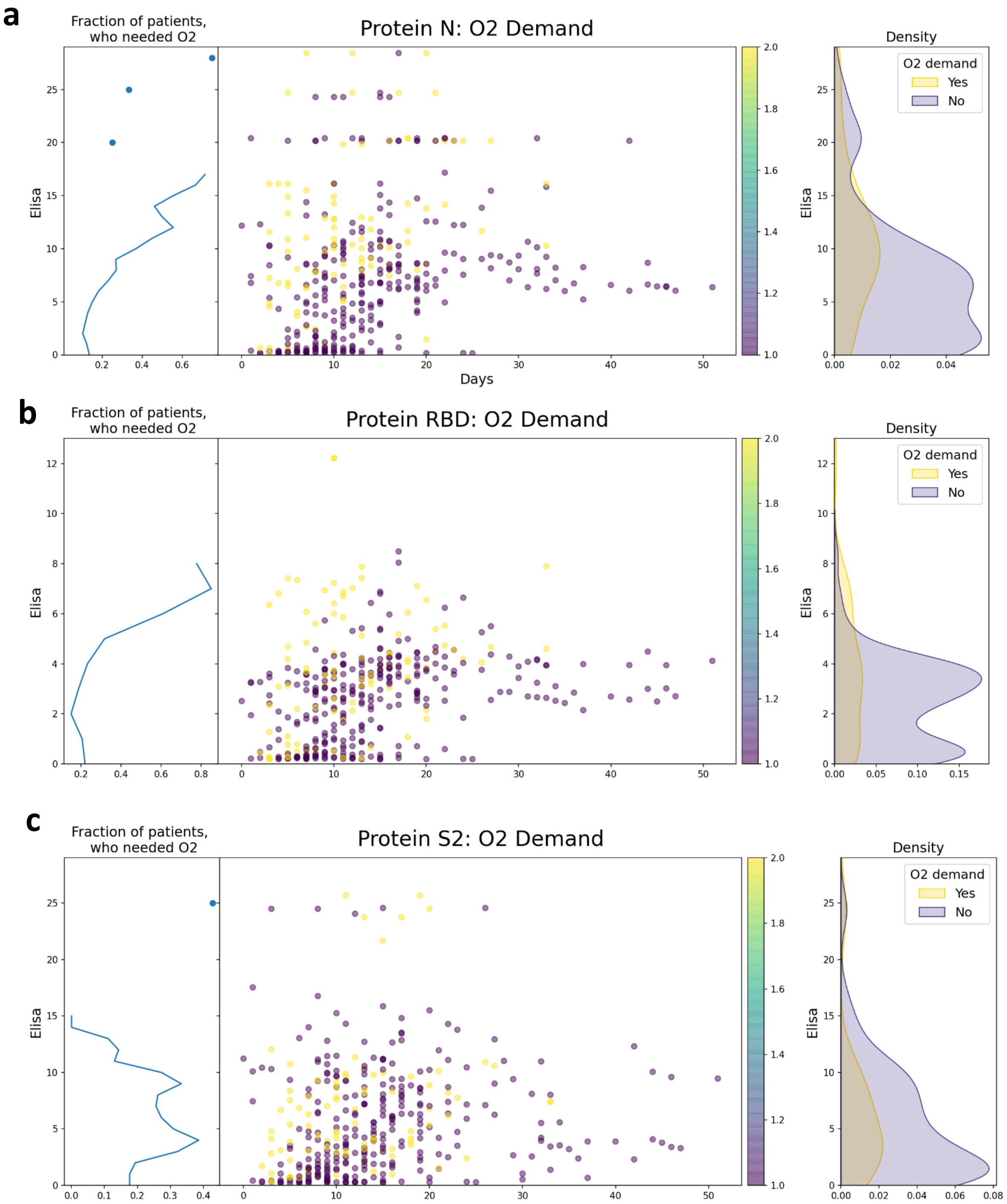

Figure S4. Growth of specific IgG antibodies in COVID-19 patient serum to the conformational N (a), RBD (b) and S2 (c) SARS-CoV-2 epitopes within 50 days among the patients who needed oxygen support and those who did not. At the scatter plots in the middle, violet dots correspond to patients requiring no additional oxygen supply, yellow – to patients who used at least nasal prongs. Line graphs on the left side illustrate fractions of patients with oxygen issues for each level of antibodies. The density plots on the right side show the density of ELISA values for the corresponding groups.

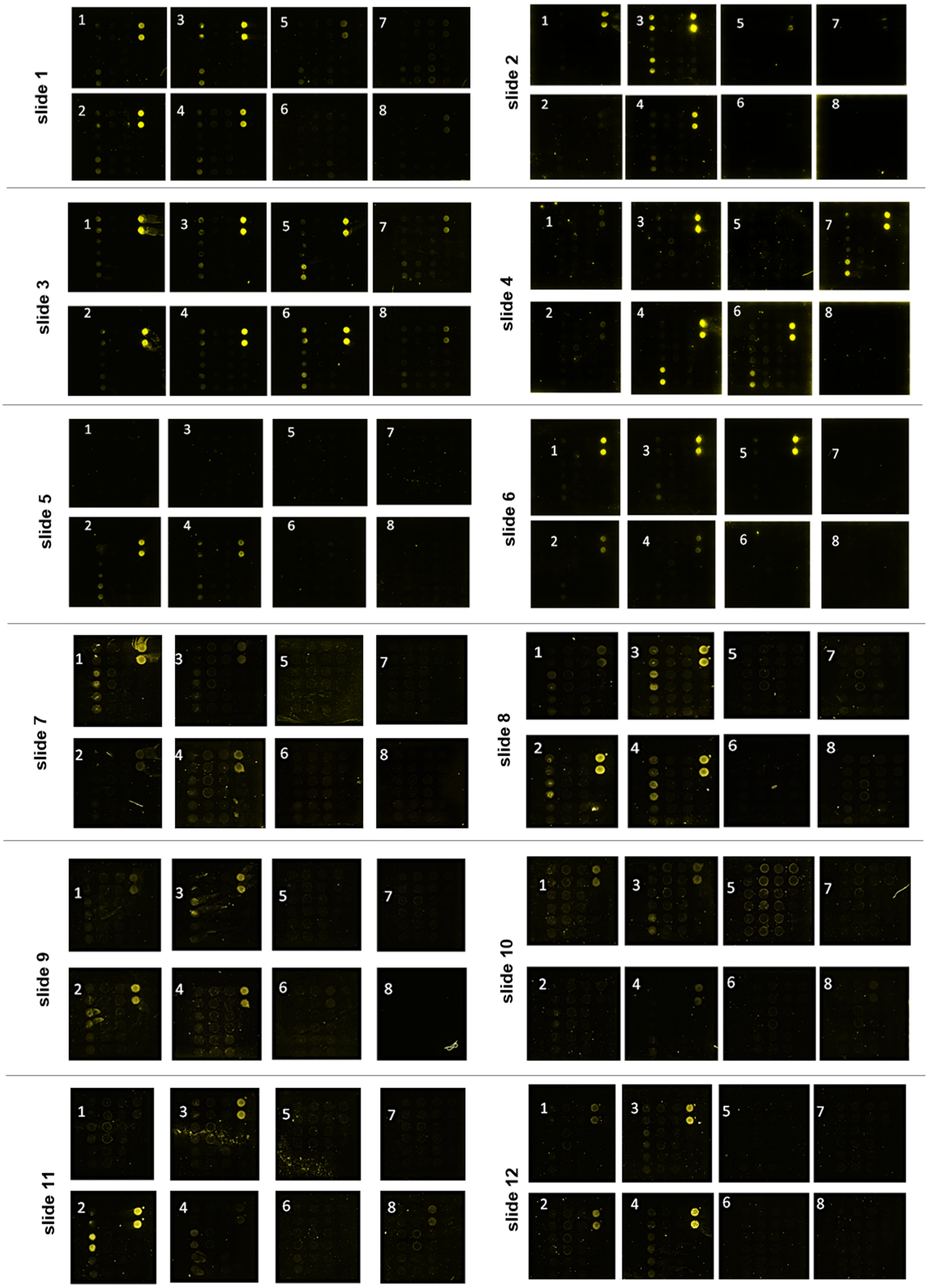

Figure S5. Examples of the array scans. Each array enabled profiling eight samples against ten SARS-CoV-2 proteins and two controls. The details are provided in Fig. S6

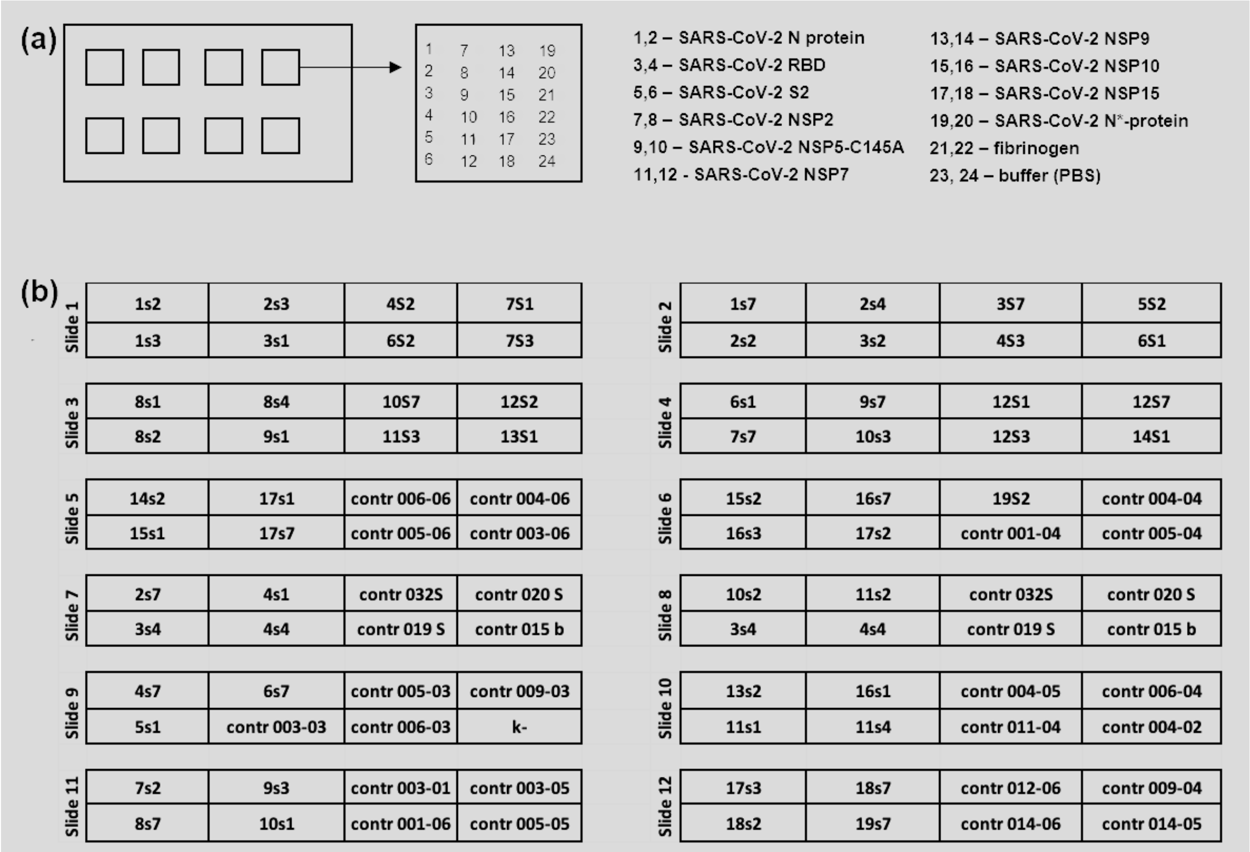

Figure S6. Design of the microarray slides. (a) Schematic representation of each array with eight square working areas, each containing 8 immobilized SARS-CoV-2 proteins in two replicates and two controls. (b) Codes of samples shown in Fig. S4. For example, 1S2 means: "1" – code of the patient in our data base; "S" – serum; "2" – second time point of the taking serum from the patient. S1 - time of admission to the hospital, S2 - after 48 hours of hospitalization, S3 - after 7 days, S7 - time of discharge from the hospital

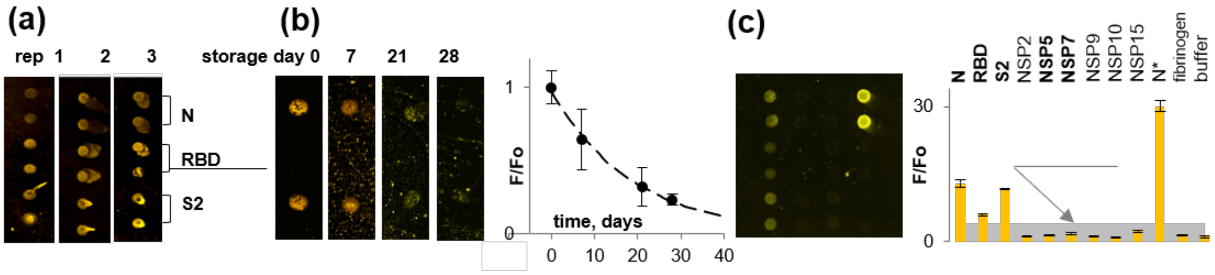

Figure S7. Microarray scans: variations. (a) Three biological repeats (two technical repeats for each protein) were performed for a single serum sample and analyzed immediately after preparation. (b) A single serum sample was analyzed using four identic arrays that have been fabricated in parallel immediately after array fabrication or 1/3/4 weeks after fabrication. The arrays were stored at +4°C between measurements. (c) Variations between the replicates are shown as error bars in the normalized signal histogram

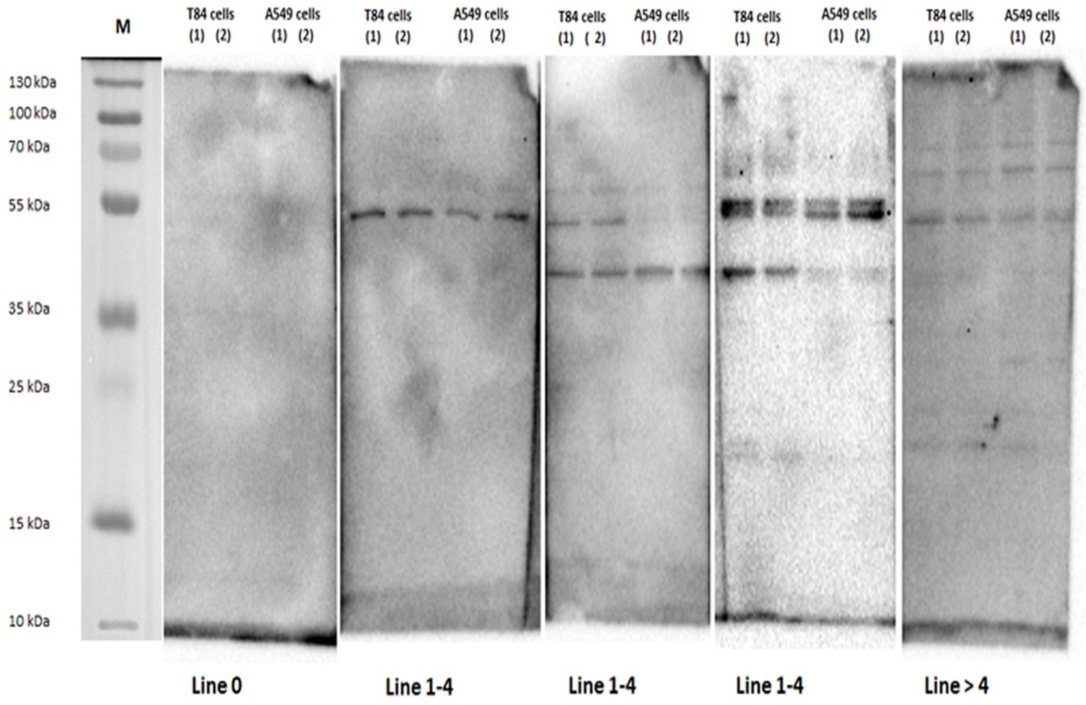

Figure S8. Examples of Western blot with antibody recognition of human proteins of two cell lines: A549 and T84. The production was performed in two technical replicas. The analysis of the obtained results was carried out based on the number of proteins bands recognized by antibodies
